# Hope and Acceptance at the End of Life: Impact of Spirituality on Patients in Palliative Care

**DOI:** 10.7759/cureus.95249

**Published:** 2025-10-23

**Authors:** Lutenio Junior, Inês Rosendo, Nuno Sena de Barros, Cátia Solis, Diana Rato, Vanessa Correia, Raquel Cruz Nunes, Inês Reis, Guilherme Ramalho

**Affiliations:** 1 Nautilus Family Health Unit, Unidade Local de Saúde do Baixo Mondego, Figueira da Foz, PRT; 2 Faculty of Medicine, Universidade de Coimbra, Coimbra, PRT; 3 Family Health Unit Coimbra Centro, Unidade Local de Saúde de Coimbra, Coimbra, PRT; 4 Baixo Mondego Community Palliative Care Support Team, Unidade Local de Saúde do Baixo Mondego, Coimbra, PRT

**Keywords:** acceptance, end of life, hope, palliative care, spirituality

## Abstract

Introduction: Palliative care aims to improve the quality of life of patients with serious illnesses. In this type of care, spirituality is placed alongside other dimensions: physical, psychological, and social. The end of life brings confrontation with dilemmas such as "none of this makes sense" or "was it worth it?" The acceptance or rejection of the journey presents spirituality as a dimension that accompanies the person in their existence. Interventions that allow for a review of life help facilitate reflection on regrets, unresolved issues, and an understanding of the legacy that generates comfort and dignity. The objective of this research was to evaluate the evolution of hope and acceptance among patients visited by the Palliative Care Community Support Team from Baixo Mondego (PCCST-BM) in Portugal.

Materials and methods: A prospective, controlled, non-blind, longitudinal research study with an intervention, featuring pre- and post-evaluation. The intervention group (IG) corresponded to patients referred to the PCCST-BM, and the control group (CG) corresponded to selected users registered at the Nautilus Family Health Unit (FHU-N) or the Coimbra Centro Family Health Unit (FHU-CC), with severe pathology, comorbidities, and a reserved prognosis. PCCST-BM patients could not have had previous contact with palliative care or have been the subject of prior home visits. In the IG, the questionnaire was administered twice: during the first home visit and the second time after 14 days. In the CG, the questionnaire was administered only once. The questionnaire included sociodemographic and clinical data, the Herth Hope Index scale - Portuguese version (HHI-PT), and open-ended questions. Descriptive and inferential quantitative and qualitative analyses were performed.

Results: Each group consisted of 10 patients, for a total of 20. The results showed a significant improvement in hope among patients in the IG (p = 0.03). The average hope between home visits also increased, reaching values similar to the CG on the second visit, despite functional decline. In the qualitative assessment, patients in the IG reported greater acceptance and positive feelings after fourteen days.

Discussion and conclusion: The follow-up carried out by the team showed improvement in hope and acceptance, with results similar to those of a specific home-based intervention study conducted in Portugal, suggesting that this approach is helpful in this context. The small sample size due to the exclusion of patients with clinical deterioration and the variable number of home visits were relevant biases. The existence of only a few studies in this area highlights the importance of further research with larger samples and other tools.

## Introduction

Palliative care aims to improve the quality of life for patients with severe or incurable diseases through a multidisciplinary approach that entails the collaborative work of different areas of healthcare, focusing on the prevention and relief of suffering [1]. This care addresses not only the treatment of physical symptoms but also psychosocial suffering, grief, family support, and spirituality [2]. Therefore, as with physical, psychological, and social dimensions, spirituality is also a fundamental dimension of being human [1]. The end of life presents itself as a dilemma, bringing with it the confrontation between "none of this makes sense" and "was it worth it?" That moment of integration or refusal of the journey refers to spirituality as the dimension that accompanies the person throughout their existence. Let us clarify that the terms "religiosity" and "spirituality" are often associated; however, the latter has a broader meaning. The acceptance of the legacy that each of us leaves to those who remain and the hope that floats in the last days of life are fundamental in this time of synthesis and questioning [2].

Spirituality can be defined as an existential motivation, a personal perspective that is complex, multidimensional, and integrative of the entire human experience. It involves processes of reflection, questioning, contemplation, and meditation [1]. The search for meaning and/or purpose is the essential motivation of life; the definition of this "meaning" is unique and specific to each person, based on their values and beliefs and influenced by their personal experiences [3]. The role of spirituality in this search is intrinsically linked to the fact that it is viewed as part of the human experience that seeks meaning and as a source of help that transcends immediate experience, restoring hope [1,4]. We can consider hope as a subjective phenomenon, but one that can benefit health and human existence [5]. It is not an escape from the present moment, but rather the ability to attribute meaning to experiences and find purpose in adversity [1,4].

In Portugal, family health units (FHUs) are functional units that operate within health centres, focusing on primary healthcare. These include medical and nursing consultations with a predefined range from 15 to 30 minutes [6]. There is no standard or established norm to address spirituality, hope, or acceptance in clinical practice in primary healthcare.

A Palliative Care Community Support Team (PCCST) provides care at home, allowing patients to remain in their familiar surroundings. It consists of a multidisciplinary team of doctors, nurses, psychologists, social workers, and other professionals who work together to promote well-being and quality of life to prevent suffering for patients, their families, and caregivers. The team's support for patients and family members is not only provided during home visits but also by telephone. These teams are part of the National Palliative Care Network within the National Health Service, alongside Palliative Care Units and Intra-Hospital Palliative Care Support Teams. As a rule, community teams are assigned to a specific geographical area, responding to patients in that region [7]. Home visits do not have a pre-established duration, as this varies according to the patient's needs and clinical condition. During home visits, besides the patients' physical and medical needs, their values, beliefs, religiosity, and spirituality are also addressed. The approach to these topics can be supported by tools such as the Hope Scale for assessing hope.

The approach to spirituality is distinctive, but it should always be carried out consciously by the entire multidisciplinary team, respecting each patient's sense of meaning in life and individual beliefs [8,9]. The importance of addressing spirituality in palliative care is therefore transversal to the entire team, i.e., doctors, nurses, social workers, and psychologists, in which no role is more important than the other, and the presence of each enhances the care provided by the team [9].

More than understanding spirituality as the search for meaning, it is necessary to integrate it into the provision of medical care. In addition to collecting medical history and performing clinical examinations, it is also essential in the context of palliative care to investigate and gather information about each patient's life story, spiritual beliefs, and values. This allows us to understand the meaning that each of them attributes to their faith, life, and spirituality. This individualized approach facilitates the creation of more personalized and holistic care and follow-up plans. Collecting this data allows an improvement in quality of life, a reduction of suffering, a better patient and family satisfaction, and the fostering of a more welcoming environment [10].

A study by Bandieri et al. looked at the impact of psychological well-being in palliative care and defined some essential dimensions to explore in this context: hope, as a promoter of resilience that impacts quality of life; gratitude, which promotes emotional connections between patients, staff, and families and leads to reduced stress; and acceptance, which facilitates adaptation to the current condition and reduces the fear inherent to the clinical situation [11]. Another study by Miquel et al explored the impact of these issues on patients' families and reinforced the importance of addressing aspects such as: philosophy of life, which impacts decision-making and how values are maintained or changed from the moment the disease appears; family relationships and how they can act as a support for everyone; and transcendence, which can be understood as the way values and beliefs interpret something inexplicable [12]. Finally, another study by Tanzi et al. reinforces the importance of trained teams that address these issues related to spirituality, as they promote a shift from a person-centered approach to spirituality and personal reflections to a deeper involvement between patients, family, and the support team [13].

In Portugal, a study was conducted with a specific intervention focused on hope in the context of patients at home with advanced chronic disease, whose schedule defined three moments of home contact between the team and the patient [5]. Once the initial approach has taken place, the second moment should occur after 15 days and the third after one month [5]. The intervention was based on a model in which hope is seen as something that can only be interpreted in relation to each person's personal journey. The role of healthcare professionals was to help each person on this journey in search of hope. The approach to hope taken during home visits covered several aspects: positive expectations, personal qualities, spirituality, goals, comfort, help/care, interpersonal relationships, control, legacy, and life review. In this individualized intervention, the three home visits were conducted by a nurse [5]. The duration of each visit ranged from 90 minutes to two and a half hours, depending on the patient's needs and preferences. A family member was allowed to be present if desired, which occurred in half of the situations. All participants underwent the same number of sessions, and the objectives were predefined for each one [5]. The first session aimed to awaken self-perception of hope and the ability to express it verbally. This session included the analysis of a video in which terminally ill patients discussed, together with their families, the importance of keeping hope alive. This allowed participants to identify with the people in the video and shape their view of their ability to perform tasks. In the second session, the focus was on expressing feelings related to the illness and strengthening the ability to perform tasks that promote hope. Several tasks were taken to this end: gratitude exercises, therapeutic and forgiveness letters (addressed to specific individuals), the creation of a hope album (to recall memories of the past), and the creation of a hope kit (containing objects of sentimental and inspirational significance). Finally, the third session was aimed at transcendence. This sought to alleviate the suffering caused by the progression of the disease through teaching and training in relaxation using mental images. The idea was to strengthen each person's ability to deal with their situation by drawing up a daily routine plan. Thus, all the techniques learned were integrated into everyday life. The study used the Herth Hope Index (HHI) scale and showed that the patients targeted by this intervention experienced an increase in hope, comfort, and quality of life [5].

The holistic approach in medicine, particularly in the context of palliative home care teams, which evaluates the person as a whole, promotes greater acceptance and empathy, and allows patients' spiritual needs to be addressed and understood [14]. Interventions that enable patients to review their lives consequently lead to reflection on their regrets, unresolved issues, and understanding of their legacy, which can generate comfort and dignity in end-of-life situations [15]. This type of intervention is expected to increase the sense of hope, decrease depression, and improve quality of life [16,17]. As demonstrated in other studies, the hope approach is a valuable strategy for promoting healthy and meaningful personal relationships at the end of life [17-20]. The use of the HHI scale in association with other tools, such as the McGill Quality of Life scale, stimulated an increase in these topics in reassessments [21].

The fact that there are still few studies on this subject, particularly in Portugal, where most published work has been conducted with specific interventions, led us to question the impact of addressing these issues in the context of usual care provided by home-based palliative care teams. Therefore, the objective of this research is to assess the evolution of hope and acceptance among patients visited by the PCCST from Baixo Mondego (PCCST-BM) and to understand the impact of its usual intervention on the care provided.

## Materials and methods

Study design

This prospective, controlled, non-blind, longitudinal study aimed to evaluate the evolution of hope and acceptance in patients with severe pathologies and poor prognosis, without prior contact with palliative care. The population was divided into two groups: an intervention group (IG), which received home visits from a palliative care team, and a control group (CG), composed of patients with a similar clinical profile, followed in primary care. The intervention included the administration of the Portuguese version of the HHI (HHI-PT) and the exploration of open-ended questions about resolving personal issues and associated feelings.

The primary outcome was the evolution of hope, measured quantitatively by the HHI-PT, and acceptance, assessed qualitatively by content analysis. As secondary outcomes, complementary clinical and emotional indicators were considered: pain intensity (Visual Analog Scale - VAS), fatigue level (ordinal scale from 0 to 3), functional status (Karnofsky Performance Status - KPS), and risk of depression (PHQ-2).

To this end, authorization was requested to use the HHI-PT. It was requested authorization by the Ethics Committee of the Faculty of Medicine of the University of Coimbra, which was favourable on December 23, 2024. This study was registered at clinicaltrials.gov (ID NCT07113899). The authors had no conflicts of interest.

Selection of participants

The sample consisted of two groups: the IG, which corresponded to patients in the PCCST-BM network, and the CG, which corresponded to selected patients who were registered at the Nautilus Family Health Unit (FHU-N) and the Coimbra Centro Family Health Unit (FHU-CC) with severe pathology, comorbidities, and a reserved prognosis. 

The selection of PCCST-BM patients was carried out considering only new patients referred to the team who had not yet been visited at home by the team at the time of the study (i.e., referrals made between January and May 2025). Thus, the first application of the questionnaire was conducted during the initial visit. The questionnaire was administered a second time after a period of 14 days. Regarding patients from the FHU-N and FHU-CC files, patients with severe pathology, comorbidities, and a reserved prognosis with the same diagnoses as the patients in the IG were selected by invitation, identified through a search of the principal pathology codes in the IG patients, and subsequently verified for the disease status in the clinical records. The questionnaire was administered to these patients once.

As soon as the second questionnaire was administered to a patient in the IG, 14 days after the first questionnaire was administered, and data collection was completed, we proceeded to select a new patient to join the CG and administer the questionnaire. This strategy allowed us to maintain a balanced number of participants in both groups until the end of data collection. In both groups, there were no refusals to participate in this study. Six patients were selected from the FHU-N and four patients from the FHUC-CC. These two units cover the territories of two different local health units: Coimbra and Baixo Mondego (this territory corresponds to Figueira da Foz, where the FHU-N is located). As for the patients in the IG, both belonged to these same two territories, as the community team operates in the territory corresponding to these two local health units. The IG consisted of ten patients, six from the area corresponding to Figueira da Foz and the remaining four from Cantanhede, which is part of the Coimbra Local Health Unit. Thus, we maintained territorial homogeneity between the groups. 

In this study, the clinical consultation at the FHU was conducted by the doctor, who also administered the questionnaire. These consultations were scheduled to last between 20 and 30 minutes.

The majority of the home visits conducted by the community team had a doctor, a nurse, and a social worker present. The questionnaire was administered by the doctor, nurse, or with the help of both. The duration of the home visit varies, as does the time available to address spirituality. As a general rule, the topic of spirituality is not typically addressed in consultations conducted at FHUs. However, in palliative care, spirituality is addressed during the clinical interview with the patient or their family. The patient's values, beliefs, and religion were questioned. The strength of these beliefs was also assessed, whether they remain unshaken in the face of the clinical condition or whether they changed in that context. Other aspects were questioned when addressing spirituality: if this belief influenced decision-making at that moment and the importance the patient gave to their faith.

Informed consent was obtained during the home visit by PCCST-BM. As for FHU-N and FHU-CC, informed consent was obtained during the consultation, either in person or by telephone. When received by telephone, it was signed in writing at the next in-person consultation. The responses to the questionnaire were stored in a protected Excel spreadsheet and were only kept on file until the statistical analysis and results were obtained.

The inclusion criteria for the IG were to be part of the PCCST-BM network, with referrals made during the defined data collection period, or to be a user of FHU-N or FHU-CC with severe pathology, comorbidities, and a reserved prognosis. Exclusion criteria for both groups were being under 18 years of age, pregnancy, an altered state of consciousness or dementia, and a low educational level that would compromise the quality of the answers given in the questionnaire. Another exclusion criterion in the IG was having had any previous contact with the team, meaning that these patients could not have been part of this network previously and could not have received any home visits from Palliative Care before the visit when the questionnaire was first administered.

Concerning conditions and funding, the individuals' participation was voluntary, and there was no financial remuneration.

Data collection

The questionnaires were administered in both groups over a period of five months: from January 1, 2025, to May 31, 2025.

In the IG, the questionnaire was administered twice: the first time during the initial home visit and the second time during another home visit 14 days later. In the CG, the questionnaire was administered once. The minimum sample size required as a target (25 participants per group) was calculated on clincalc.com with an alpha of 0.05 and power of 80%, based on a previous study [5]. 

The questionnaire covered sociodemographic data (age, gender, marital status, and education), clinical data, the HHI-PT, and open-ended questions. The clinical data collected included the primary diagnosis, secondary diagnoses, functional status (assigned according to the KPS scale), pain (defined by the application of the VAS), fatigue (on a scale of zero to three, where zero corresponded to “absent,” one corresponded to “mild,” two corresponded to “moderate,” and three corresponded to “severe”) and depression screening scale PHQ-2.

For a better study of the primary diagnoses obtained, these were grouped by the principal investigator. In situations where the primary diagnosis could be included in more than one group, each case was analyzed individually to understand the clinical problem of each patient and the etiology of each case, thus integrating it into the most appropriate diagnostic group. Therefore, the following groups were defined: “autoimmune pathology,” which included primary diagnoses of myasthenia gravis, multiple sclerosis, myelitis, and polymyositis; “neoplasia,” which included diagnoses of paraganglioma, squamous cell carcinoma, genital cancer, lung adenocarcinoma, colon cancer, breast cancer, pancreatic cancer, and prostate cancer; “degenerative disease,” which included diagnoses of amyotrophic lateral sclerosis and osteoarticular pathology; “hematological pathology,” which included the diagnosis of multiple myeloma; and “hepatic pathology,” which included the diagnosis of “chronic liver disease.” The main diagnosis was decisive for matching between groups and the selection of patients by invitation.

Authorization was requested to use the HHI-PT scale, which was granted. The validated Portuguese version of the scale was used [22].

During the first home visit by PCCST-BM and during the consultation at FHU-N or FHU-CC, three open questions were asked: “Is there anything in your life that needs to be resolved?” “Would you like to resolve this issue?” and “How do you think you would feel if you resolved this issue?” During the second PCCST-BM home visit, two weeks later, the three questions mentioned above were asked, along with a fourth question: “How do you feel now that the situation has been resolved?” The aim was to characterize and analyze the evolution in the answers given at these two moments.

**Figure 1:**
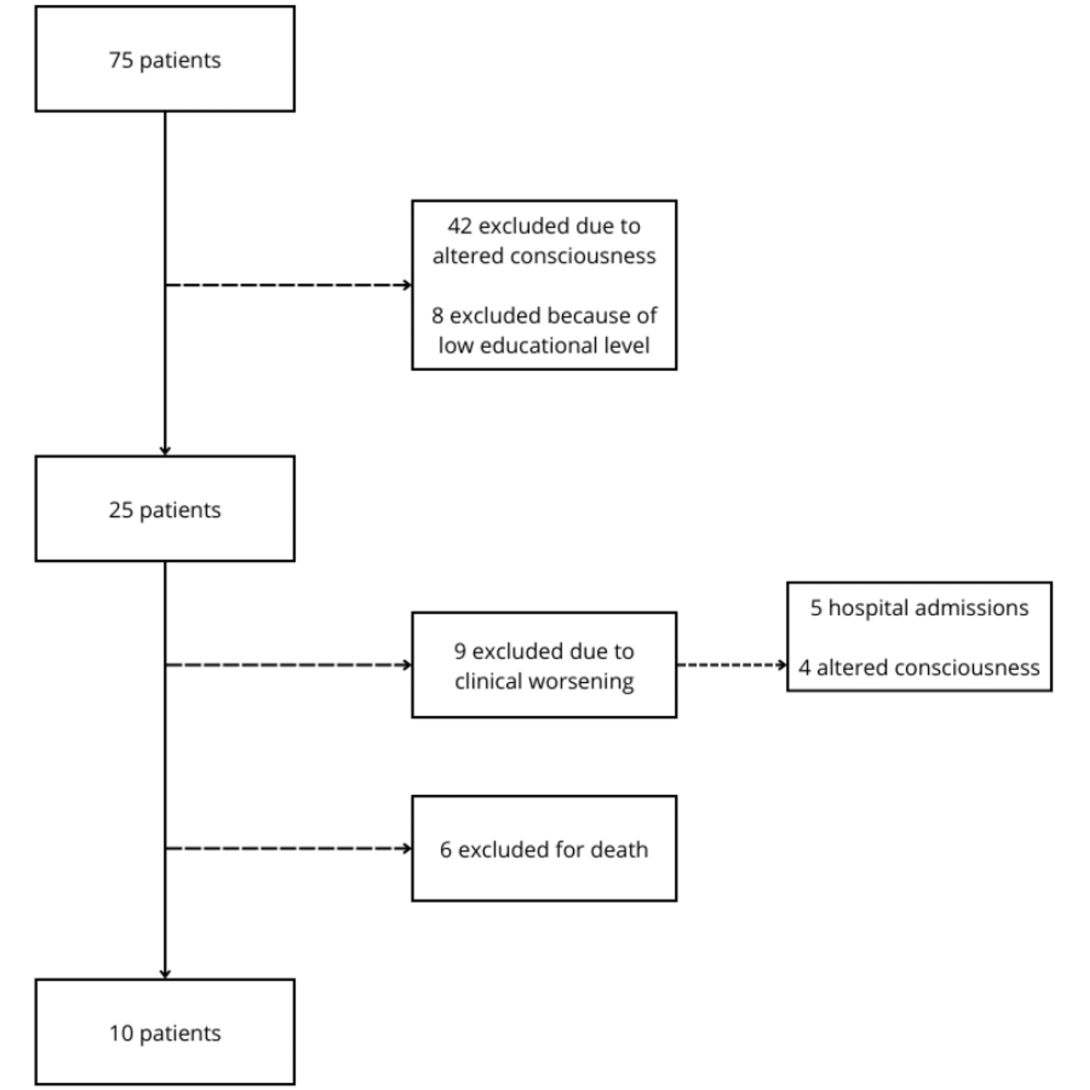
Flow chart of the selection of participants in the intervention group

During the period defined for data collection by PCCST-BM, 75 referrals of new patients were received. The patients’ family doctors made all these referrals. Of the 75 referrals, 42 patients were excluded due to changes in their level of consciousness, and eight were excluded due to low educational attainment. Of the remaining 25 patients selected, 15 were excluded after the first questionnaire was collected: nine due to worsening clinical condition, which meant they met the exclusion criteria, and six due to death. Of the nine patients whose clinical condition deteriorated, five were readmitted to the hospital, while four experienced changes in consciousness at home that would compromise their responses in a second application of the questionnaire. Thus, 10 patients remained (Figure 1).

Instruments

The functional status of each individual was assigned by the researcher, according to the KPS, at the time of the questionnaire application. On this scale, the researcher classified patients on a percentage scale from 0 to 100 [23].

Pain was classified using the VAS. Patients assigned a score from 0 to 10 to classify the intensity of their pain. A score of 0 corresponds to no pain, and a score of 10 corresponds to maximum pain intensity.

To study each patient's fatigue, they were asked about its presence and asked to respond according to the following scale: "zero" corresponded to no fatigue, "one" corresponded to mild fatigue, "two" corresponded to moderate fatigue, and "three" corresponded to severe fatigue.

To assess the risk of depression, PHQ-2 was used. This scale consists of two questions focused on the absence of interest or pleasure and feelings of sadness in the last two weeks. The score was assigned by the patient, in each question, on a scale of zero to three, depending on the frequency with which these situations occurred. A final score of three or higher indicated a high probability of depression.

The HHI-PT scale was used to study hope. There are two validated versions of the HHI-PT scale in Portuguese. We excluded one version because it was recommended to assess the caregiver performance in the nursing area. The version selected includes nine questions with four possible answers, which we decided to score as “strongly disagree” (1), “disagree” (2), “agree” (3), and “strongly agree” (4). This scoring is intended to provide greater clarity in the interpretation of the data obtained. This scale enables the assessment of hope through elements such as faith, optimism, ability to plan for the future, and connection with others. Cronbach's alpha coefficient (0.873) attests to the good internal consistency of this tool [22].

Data analysis

An Excel database (Microsoft Corp., USA) was created. IBM SPSS Statistics for Windows, version 30.0 (released 2024, IBM Corp., Armonk, NY) was used to process and analyze the data. 

In the initial phase, a descriptive analysis of both samples was performed. 

To assess potential discrepancies between the groups and determine differences before and after the PCCST-BM intervention, we performed an inferential analysis. We used the Chi-square test and Fisher's exact test to compare nominal variables (gender, marital status, education, and diagnosis) between the groups. Next, we verified the normality of the distribution of continuous numerical variables (age, functional status, pain, and HHI-PT) by performing the Shapiro-Wilk test. All variables were normally distributed, so we used parametric tests, namely, the T-test, to compare the means of independent variables (intervention and control groups) and paired variables (before and after the intervention). Scalar variables (fatigue) were evaluated using the Wilcoxon test. 

A qualitative analysis of the responses to the final open-ended questions was performed by categorizing them. The perceptions and feelings reported were coded in the categories by content analysis by two independent researchers. The results were compared, and any discrepancies were analyzed and resolved by consensus, ensuring reliability and methodological rigor.

## Results

The total sample for both groups was 20, with 10 users in each. There was an equal distribution between males and females (n = 10; 50%). Table 1 shows that most individuals were married (n = 13; 65%), and their education levels ranged from 4th grade to a bachelor's degree. The primary diagnoses were grouped, with a predominance of the Cancer group (n = 12, 60%). Regarding age, the average was 69.85 ± 10.30. There were no significant differences between the CG and IG in terms of sociodemographic variables or primary diagnoses (Table 1).

**Table 1:** Descriptive statistics of sociodemographic variables and diagnoses in the intervention and control groups and results of the inferential analysis comparing the groups The data has been represented as number and percentage. *The significance level is p < 0.05 (significant) using the Chi-square test. **The significance level is p < 0.05 (significant) using Fischer's exact test.

		Number (total)	Percentage (total)	Number (control)	Percentage (control)	Number (intervention)	Percentage (intervention)	p
Gender	Female	10	50	6	60	4	40	0.66*
	Male	10	50	4	40	6	60	
Marital status	Married	13	65	6	60	7	70	0.08**
	Single	0	0	0	0	0	0	
	Separated	0	0	0	0	0	0	
	Widowed	2	10	2	20	0	0	
	Divorced	3	15	0	0	3	30	
	Common-law marriage	2	10	2	20	0	0	
Education	4th grade	8	40	5	50	3	30	0.61*
	7th grade	1	5	0	0	1	10	
	9th grade	6	30	3	30	3	30	
	12th grade	3	15	2	20	1	10	
	Bachelor's degree	2	10	0	0	2	20	
	Master's degree	0	0	0	0	0	0	
	Doctorate degree	0	0	0	0	0	0	
Diagnosis	Autoimmune disease	4	20	2	20	2	20	1.00*
	Cancer	12	60	6	60	6	60	
	Degenerative pathology	2	10	1	10	1	10	
	Hematological pathology	1	5	0	0	1	10	
	Liver disease	1	5	1	10	0	0	

Regarding functional status, it was observed that patients in the CG had an average score of 62. By contrast, patients in the IG had a lower average score of 46 at the beginning, followed by a significant decline in functional status as indicated by the second application of the questionnaire, with the average score decreasing to 42. There was a statistically significant difference between the CG and the second assessment of the IG (p = 0.01) and between the first and second assessments of the IG (p = 0.04). (Table 2).

**Table 2:** Descriptive statistics of the clinical variables and results of the inferential analysis comparing the groups. CG: control group; IG: intervention group; KPS: Karnofsky Performance Status; VAS: Visual Analog Scale; HHI-PT: Herth Hope Index The data has been represented as number (minimum and maximum values obtained) and mean±SD. *The significance level is p < 0.05 (significant) using the Shapiro-Wilk test.

	CONTROL	N (minimum and maximum obtained)	Mean±SD	INTERVENTION BEFORE	N (minimum and maximum obtained)	Mean±SD	INTERVENTION AFTER	N (minimum and maximum obtained)	Mean±SD	p	p (CG and IG later)	p (between IG)
Functional status (KPS)		50 - 80	62.00±12.29		30 - 60	46.00±10.75		30 - 60	42.00±11.35		0.01*	0.04*
Pain (VAS)		0 - 9	4.10±3.60		0 - 9	3.90±3.54		0 - 10	4.30±3.34		0.90*	0.65*
Hope (HHI-PT)		23 - 36	29.60±4.86		14 - 35	26.20±5.92		23 - 36	30.20±5.01		0.79*	0.03*

Regarding self-perception of pain, there was no significant difference between groups or between the first and second assessments; the CG average was 4.1, while the IG average was 3.9 in the initial evaluation and 4.3 in the second assessment (Table 2).

Regarding hope, on a maximum possible score of 36, the HHI-PT showed that the CG obtained an average of 29.6 in their responses. For the IG, a significant improvement was observed in the average responses from the first to the second assessments, which rose from 26.2 to 30.2 (p = 0.03), and in the end did not differ significantly from the CG (p = 0.79) (Table 2).

Regarding fatigue, the median was 2 for all groups, with no significant differences between groups and before and after the intervention (Table 3).

**Table 3:** Fatigue analysis CG: control group; IG: intervention group The data has been represented as median and percentile. *The significance level is p < 0.05 (significant) using the Wilcoxon test.

Group	Median	Percentile		p (CG and IG later)	p (between IG)
		25	75		
Control	2	2	3	0.34*	0.48*
Intervention before	2	1	2.25		
Intervention after	2	1.5	3		

In the screening for depression, the risk of depression was present when the sum of the two questions asked by the PHQ-2 scale was equal to or greater than three. The results in the following table show that there were more people at risk of developing depression in the CG with no significant differences. In turn, in the IG, in both assessments, the majority of patients had no risk of depression, with no significant differences (Table 4).

**Table 4:** Depression screening – PHQ2 CG: control group; IG; intervention group The data has been represented as number and percentage. * The significance level is p < 0.05 (significant) using the T-test for independent variables (intervention and control groups). ** The significance level is p < 0.05 (significant) using the T-test for paired variables (before and after the intervention).

Group	PHQ-2				p (CG and IG after)	p (between IG)
	No risk of depression		Risk of depression			
	n	%	n	%		
Control	4	40	6	60	0.37*	
Intervention before	8	80	2	20		0.07**
Intervention after	7	70	3	30		

To complement this study, the participants in both groups were asked to answer the following questions: “Is there anything in your life that needs to be resolved?”, “Would you like to resolve this issue?” and “How do you think you would feel if you resolved this issue?”. In the case of the IG, a fourth question was also asked during the second home visit: “How do you feel after the situation has been resolved?”.

In the CG, 60% (n = 6) of patients reported not identifying any unresolved issues, while 40% (n = 4) responded affirmatively that they had some unresolved issues. Of these four patients, three indicated that they would like to resolve this pending issue, while the other participant expressed doubts about his intention.

In the IG, 80% (n = 8) of participants indicated that they had some unresolved issues in their lives. Of these patients, seven stated that they would like to resolve the issue in question, while one expressed doubts about the question. At the time of the second assessment, the number of patients who responded affirmatively that they had issues to resolve decreased to half of the responses found in the first application of the questionnaire, standing at 40% (n = 4). The four participants responded that they would like to resolve this issue.

Regarding the question “How do you think you would feel if you resolved this issue?”, the participants reported expected feelings of happiness, fulfillment, comfort, and tranquility. Their testimonies demonstrated a willingness to take action to correct something that bothered them or to do something in life to achieve a sense of peace with themselves. Two participants reported being unable to express their feelings when resolving what bothered them because of the difficulty of imagining this scenario as real. Regarding the question “How do you feel after the situation is resolved?”, there were reactions of faith, inner peace, freedom, love, satisfaction, and confidence. The team's intervention led to greater acceptance by patients through various approaches: trips to Fátima (a religious city), family conferences, or establishing contact with previous disagreements within and outside the family, therapeutic listening, reframing events and people, and introspective interviews addressing the life led and legacy for those left behind (Table 5).

**Table 5:** Results of the qualitative analysis of responses to open-ended questions

How do you think you would feel if you resolved this issue?		
Reactions	No.	Reports
Happiness	6	“Happier”; “Content”; “With greater well-being and spirited ”; “More excited”; “More complete”; “Joyful”
Tranquility	2	“At peace, calm”; “I would feel much better, more peaceful. This situation makes me sad, and I would feel happier if it were resolved.”
Fulfilment and freedom	3	“Lighter”; “Free, nothing would weigh me down anymore”; “I feel free and fulfilled”
Comfort	2	“More comfortable, but there are always things to plan”; “Well, more comfortable with life”
Relaxation	1	“Relaxed”
Strength	1	“With more strength to fight”
Don't know	2	“I don’t know”; “I can’t imagine that happening, it’s too difficult”
How do you feel after the situation has been resolved?		
Reactions	No.	Reports
Freedom	1	“I feel that nothing holds me back anymore... forgiving allows us to move forward.”
Inner peace	2	“I feel calmer after visiting me. And I feel good after talking... talking and solving problems helps.” “At least I tried to talk, I did my part.”
Faith	2	“After going to Fátima, I feel encouraged; going there helps me.” “I feel accompanied, stronger.”
Love	1	“I feel loved and that people care about me.”
Satisfaction	1	“I am satisfied with what I have done and built, but now I just want to think about my next project.”
Confidence	1	“More confident about the future, days will be better.”

## Discussion

In this study, it was possible to verify an increase in the hope of patients in the PCCST-BM network between the first home visit and the visit made two weeks later. This increase in hope, in situations where, due to the expected progression of the disease, there may have been a deterioration in the patient's clinical condition (as verified, for example, by the average values obtained in functional status between visits), demonstrates the importance of the distinct approach that palliative care provides compared to others. However, the sample was not random, so a quasi-experimental and non-randomized study could bring potential selection biases, as the most motivated patients with greater hope or potential for improvement. The selection of these kinds of patients could have been the reason for such positive results.

In other studies that examined hope and quality of life during home visits, an increase in hope was also observed. However, different tools or versions of the HHI scale were used, and the visits were conducted by nurses rather than a multidisciplinary team [5,21].

Another study conducted in Portugal, in a population of patients with advanced chronic disease, also used the same scale as our study and showed an increase in hope [5]. However, this study involved a specific intervention that took place during three fixed home visits [5]. By contrast, in our study, the visits were variable and adjusted to the overall needs of the patient. In addition, before the first home visit, a screening was carried out in which the selected patients were given the Mini-Mental State Questionnaire, their functional status was assessed according to KPS, and they were asked about the presence of uncontrolled symptoms; patients who did not pass this screening were excluded [5]. In that study, only hope, quality of life, and comfort were reassessed, and an improvement in these three topics was observed [5]. The study focused on assessing the same symptoms as our study with different tools and without reassessing all of them in subsequent visits [5]. The topics to be addressed in this study were defined before the home visits and aimed to specifically improve the areas tested and only by a nurse, unlike our study, which studied the usual intervention of the home palliative care team, by a multidisciplinary team [5]. The variability in the number and duration of visits should be taken into account when analyzing the results.

As for hope, there may be several reasons for this improvement in the IG: the duration of the palliative care team's home visit is much longer than the time of an appointment in primary health care. This time allows for therapeutic listening without the pressure of ending the consultation to see the next patient. Having the space and time to listen to others can make a big difference in the connection created between team members and patients, as well as in the patients' perception of the team's involvement in their particular case. 

Another distinct difference between community-based team care and primary health care is the dynamics of family involvement. When doing a home visit, we are not just visiting the patient. We are seeing him in their family context, observing their routines and habits in their most personal environment, as well as the dynamics between them and the other members of the family and caregivers. Family and caregivers also have more direct contact with the Support Team during the home visit, compared to the information they can provide indirectly when they go to the Health Center and report on what is happening at home. 

If we analyze the average hope values obtained in primary health care, we see that they are higher than those of the first visit carried out by the PCCST-BM. The diagnosis of serious illness, confinement at home, and loss of autonomy may justify low expectations. However, after the second visit, the values increased and exceeded those found during consultations at the health center. This shows the importance of dialogue and follow-up that the PCCST-BM provides to patients. The fact that there is only a second evaluation in the IG due to the design of the study must be taken into account when analyzing the results

When it comes to acceptance, we can see some differences. While in primary health care, we observed that 40% of patients identified unresolved issues in their lives; in the Community Support Team, this doubled to 80%. A question as personal as this requires reflection, so the person asking the question and the context in which it is requested may influence the answer. The fact that the palliative care physician asks the question in an intimate setting such as the patient's home is different from being asked by the family doctor at the health center, either because it may not provide regular follow-up or may not provide follow-up at that more specific and sensitive time, there may have time issues or even because the question is asked over the phone, as happened in one case. In addition, a change in the perspective of life may help in understanding this shift in priorities or urgency in identifying “doors to close.” Although it was common to both groups that the majority intended to resolve what was causing them discomfort, the patients, followed by the Palliative Care Team, were able to identify these situations as a greater need. The personal and clinical context in which patients found themselves can help understand these findings: a patient at the end of life with functional deterioration will undoubtedly take a different view of their experience compared to someone who still has the autonomy to go to a Health Center appointment on foot. The decrease in functional status, with reduced mobility and difficulty in performing activities of daily living, may lead to a different reflection on the life journey that has led to that point.

In both groups, most participants believed that solving what was causing them discomfort and weighing on their emotional state would be beneficial. In the IG, all participants reported an improvement in their emotional status in the reassessment after two weeks. Something common to all reports was the reference to spirituality, the transcendence expressed in faith or in each person's desire to accomplish something before death. There were reports of improved mood and acceptance because the PCCST-BM made it possible to travel to Fátima or to have a conversation with a family member or former partner. The HHI-PT directly questions faith, optimism, ability to plan the future, and connection with others; however, other topics, such as the freedom felt towards the past that each person carries or satisfaction with the legacy left behind, which were identified in the open questions, should also be studied.

Although this is difficult to measure, as it always depends on each person's subjective interpretation, the importance of approaching spirituality as a means of enhancing acceptance has already been mentioned in previous studies [10-12]. The importance of integrating the theme of spirituality into clinical practice is mentioned in a study for its contribution to patient acceptance, even making death no longer a taboo subject or associated only with negative connotations [11]. The sooner these issues are addressed for these patients, the more sustainable the doctor-patient relationship will be, and the easier it will be to reformulate certain concepts, such as hope, gratitude, or death [11].

Recent studies identify spirituality as something important among culturally and geographically diverse populations, with an impact on quality of life and end-of-life decision-making, which should be integrated into medical care [10]. Another recent study explores the plurality of the concept of hope, examining how it has helped patients and healthcare professionals in various clinical contexts to cope with unfavorable prognoses. The promotion and increase of hope were essential in achieving improvement [24]. A review found that hope rises when the patient is motivated to set specific goals, projecting alternative outcomes, promoting contact with elements where there is an emotional connection, and verbalizing desires [25].

Regarding symptoms, existing studies are scarce on this topic. Some studies report that introducing the subject of spirituality in the approach to palliative care patients can lead to a decrease in depression levels [5]. Another study conducted in Portugal evaluated patients with higher levels in the KPS (between 80% and 100%) in its intervention group, indicating a better functional state of these patients compared to those assessed in this work. This is likely because patients with uncontrolled symptoms were excluded [5].

Pain was analyzed in a "yes" or "no" dichotomy in the other Portuguese study, with most patients in the IG denying pain. In the CG, the number of patients who confirmed pain was nearly the same as the number of patients who denied it. If we consider that these data were only evaluated once, we can observe some similarities with our study, as the average values obtained in the patients' self-perception of pain were low in both evaluations and in both groups [5].

Regarding fatigue, in our study, the average value in both evaluations, across both groups, was approximately moderate in intensity. In the other Portuguese study, this question was assessed on a "yes" or "no" scale, with almost all participants reporting fatigue in both groups [5].

As for depression, in this study, the analysis was performed by answering the question of whether there was a risk of depression based on the score obtained. In the CG, there was an increase in the risk of depression (60%). In the IG, most patients did not present a risk of depression in both interventions performed. We can say that a worsening of symptoms and deterioration of the general clinical condition of this type of patient over time, hence the interest and importance of monitoring these data together with the study of hope and acceptance, which may have some influence on the levels of self-reported depression in our IG.

This study had limitations that may have affected the results. First, the sample size was smaller than initially calculated. According to the clincalc.com tool, for an alpha of 0.05 and power of 80%, with the data now obtained, 1,094 participants would be needed in each group. The complexity of palliative care patients or those with severe pathology makes it possible for their clinical condition to worsen and to die at a higher rate than in ordinary patients, which makes recruitment and retention in the study difficult. A considerable number of patients were excluded from the sample at an early stage due to changes in consciousness or neurological pathology that made it difficult to understand the questions asked and to articulate lucid answers. Even during the period between the first and second application of the questionnaire, patients were excluded due to the worsening of their clinical condition, which resulted in new hospital admissions or even death, influencing the participants in each group.

The period between the first and second home visits may also have brought some limitations. Initially, it was decided that the period would be 14 days. However, the definition of the 14-day period was a point of disagreement between family doctors and palliative care professionals. While primary health care professionals believed that two weeks would be an excessive time to collect data on patients with severe pathologies due to the risk of clinical decompensation and exclusion from the study, palliative care professionals felt that 14 days were insufficient to reassess the work they had done with patients and their families. According to these professionals, this period should be extended. After the study was completed, we believe that the two-week period is excessive and should be shortened, both because it may exclude some patients from the research and because of the work carried out by the PCCST-BM. This work begins to have an impact from the very first visit, as the topics covered only have space and time for analysis at that moment. Consultation time is also listening time. However, reducing the time between evaluations made it difficult to know the patient, their family, and their circumstances. Monitoring them from an earlier stage could also be a strategy to adopt to promote an increase in study participants.

A relevant bias in our study regarding the time between home visits was the number of visits. It should be noted that the questionnaires were collected at two different times, 14 days apart; however, patients may have been the subject of other home visits by the team between these two moments. Whether due to the worsening of the clinical condition, at the request of the family, or even because the PCCST-BM's analysis of the case motivated it, the truth is that more visits can generate closer contact between everyone, which increases hope and improves patient acceptance. In addition, these follow-up visits could have a different morphology, as the team may have also contacted the patient and family by telephone.

The number of researchers should also be mentioned. While the PCCST-BM had two professionals to administer the questionnaires, the data collection carried out at the FHU-N and FHU-CC was performed by a larger number of professionals due to the difficulty of selecting patients who met the inclusion criteria in the files of these units. In addition, questionnaires were administered in person and via telephone consultation in primary health care.

Concerning symptoms, it should be noted that patients in both groups were taking medication to control these symptoms, which was not questioned. However, the medication typically used in primary health care and palliative care differs, and the perception of symptoms may be disparate.

Regarding open questions, it is important to recognize a methodological asymmetry between groups. While both groups were asked to reflect on unresolved matters, only the IG had a second opportunity to revisit this question after 14 days. This discrepancy introduces a potential bias in the qualitative analysis, as the absence of a second measurement in the CG limits the ability to capture changes in perception over time.

Another point is that in the PCCST-BM, the researchers who administered the questionnaires were aware that a revaluation would be done and what would be asked in it. This knowledge, even unintentional, could lead to a bias in the questions and answers and a modification of the intervention itself. The alternative would be for another researcher to conduct the revaluation without the team's knowledge. Still, the logistical difficulty makes it hard to implement, mainly due to the small number of professionals with expertise in this area, and it would still not make the study completely blind.

All these points should be considered in future studies. It would be essential to ensure larger samples of participants, either by shortening the period between assessments or by increasing the period designated for data collection. Shortening the period between assessments to seven days, for example, would mean that fewer patients would be lost. About primary health care patients, extending the study to more than two FHUs would lead to a larger sample of patients with severe pathology to select, and it would also be interesting to carry out two assessments with the same number of days between them to understand differences. It would be pertinent to include the total number of home visits made by the team or the number of intermediate visits between the first and second assessments. It would also be essential to understand the type of contact: whether it was in person or by telephone. And if by telephone, whether it was only with the patient, with a family member, or with both. Increasing the number of revaluations to two or three, less spaced over time, would also be beneficial.

Finally, another critical point would be to conduct a study of consultation time. While in primary health care consultation times are already predefined according to the type of consultation, in palliative care and home visits, this point is more variable. The duration of home visits was adequate, but it would be interesting to have a record of them. We can assume that a home visit would never take less than an hour, but given that time is often greatly exceeded, it would be interesting to study this issue in greater detail.

Regarding the structure of the questionnaire, it would be interesting in future studies to explore this issue in greater depth, either with other tools or with additional questions, to understand the most critical issues for these patients. For example, greater acceptance triggered by a question of faith or religiosity or by forgiveness in a personal relationship are distinct issues. Exploring this would also allow for better targeting of the questions asked.

Despite the limitations described above, this study stands out from others due to the innovative context in which it took place. In addition, it utilized validated scales and included a CG for comparison. This work opens the possibility of exploring strategies that not only allow for a more effective approach to spirituality in palliative care but also provide helpful information for the design of future studies addressing these topics. There are still only a few studies of this type that address these issues, so this research aims to emphasize the importance of research in this area.

## Conclusions

The approach of the PCCST-BM has contributed to an increase in the hope of patients at the end of life, despite the functional deterioration that occurred in these patients. In addition, the acceptance of these patients also increased during the home care provided by the PCCST-BM.

As we see, more studies would provide some clarification. In future studies, it would be essential to reduce the time between assessments, increase the sample size, and record the number of reassessments. Even so, in patients with severe pathology, the risk of being excluded due to the worsening of their clinical condition will always be present.
